# Effects of Pep19 on adipose tissue distribution and metabolic parameters in adults with obesity: A randomized, double-blind, placebo-controlled trial

**DOI:** 10.1016/j.isci.2026.117471

**Published:** 2026-09-08

**Authors:** Davi Vantini, João Roberto de Sá, Roseli O.S. Sarni, Sonia Hix, Fabio L. Filho, Raphael Ruiz, Ricardo A. Remer, Arnon Krongrad, Luiz Felipe Martucci, Emer S. Ferro, Andrea S. Heimann, Fernando L.A. Fonseca

**Affiliations:** 1 Department of Pathology, Discipline of Clinical Analysis Centro Universitário FMABC (FMABC University Center), Santo André, Brazil; 2 Department of Medicine, Discipline of Endocrinology, Centro Universitário FMABC (FMABC University Center), Santo André, Brazil; 3 Department of Pediatrics, Centro Universitário FMABC (FMABC University Center), Santo André, Brazil; 4 Discipline of Clinical Biochemistry, Centro Universitário FMABC (FMABC University Center), Santo André, Brazil; 5 Proteimax Biotechnology Israel, 7-1 Pinchas Street, Pardes Hanna-Karkur, Israel; 6 Pharmacology Department, Biomedical Science Institute, University of Sao Paulo, São Paulo, Brazil; 7 Pharmaceutical Sciences Department, Federal University of São Paulo (UNIFESP), Diadema Campus, São Paulo, Brazil

**Keywords:** obesity, diabetes, insulin resistance, HbA1c, cardiometabolic health, central adiposity, randomized trial, intracellular peptides

## Abstract

Whether modulation of adipose tissue distribution alone can improve insulin sensitivity independently of weight loss remains unclear. In this randomized, double-blind, placebo-controlled 90-day trial, we investigated whether adults with obesity could achieve metabolic improvement through modulation of adipose tissue distribution independent of weight loss. Fifty-six participants received oral placebo or Pep19 (5 or 10 mg daily). Regional fat distribution and metabolic parameters were assessed longitudinally. Pep19 at 10 mg reduced central adiposity, as demonstrated by a reduced android-to-gynoid fat ratio and decreased abdominal skinfold thickness, without affecting body weight, caloric intake, or physical activity. Glycated hemoglobin (HbA1c) and HOMA-IR were significantly reduced, despite unchanged fasting glucose, indicating improved insulin sensitivity. These findings suggest that Pep19 promotes favorable changes in adipose tissue distribution accompanied by improvements in insulin sensitivity independent of significant weight loss, supporting further evaluation in larger and longer-term clinical studies.

## Highlights

• Pep19 improves android-to-gynoid fat distribution while preserving lean mass
• Pep19 enhances insulin sensitivity and glycemic control in obesity
• Pep19 shows a favorable safety and tolerability profile after 90 days
• Pep19 represents a promising early metabolic intervention for obesity

## Graphical abstract

**Figure:**
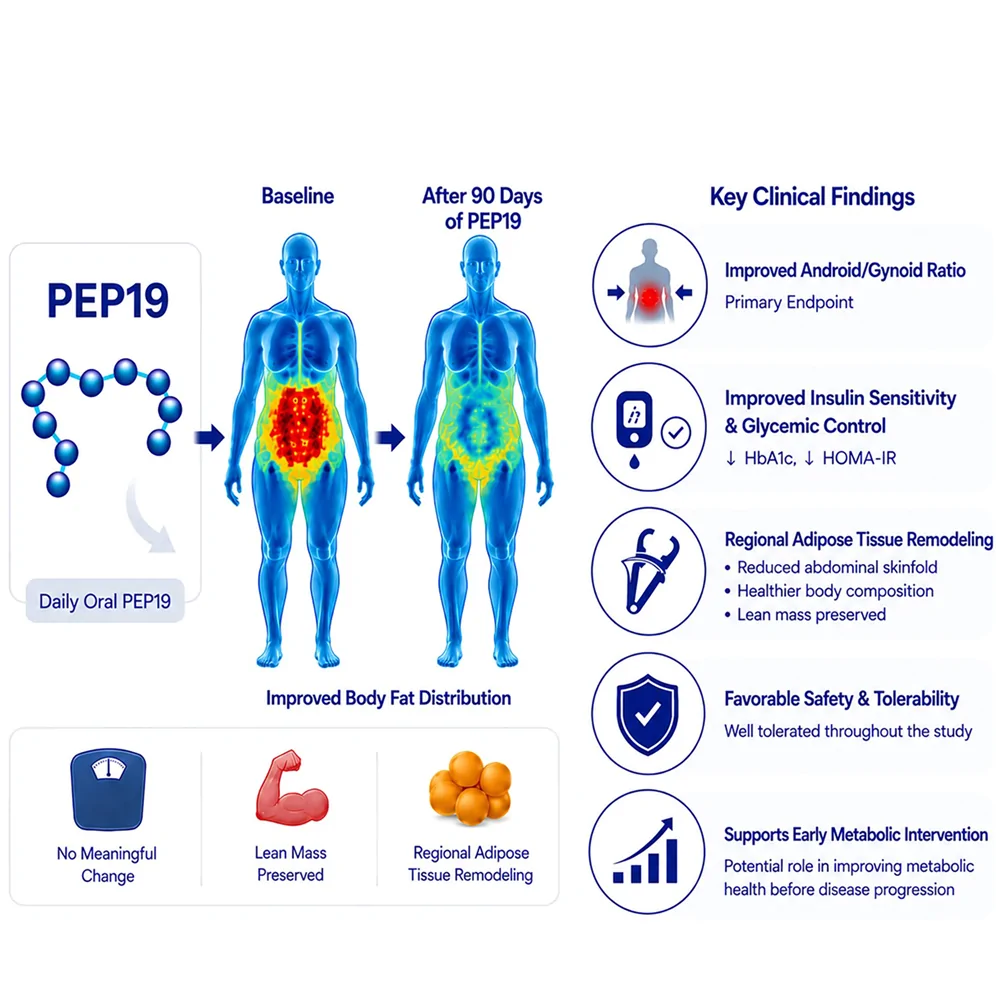

## Teaser

Health sciences; Medicine; Medical specialty; Internal medicine; Endocrinology; Therapeutics

## Introduction

Obesity treatment has historically linked metabolic improvement to reductions in total body weight.^1^ However, emerging evidence suggests that regional adipose distribution, particularly visceral adiposity that acts as an active endocrine organ rather than just a storage site for energy,^2^ may be a more direct driver of metabolic risk than body mass alone.^3^ Among the pathophysiological drivers of obesity, visceral fat together with chronic hyperglycemia play a central and synergistic role.^3^^,^^4^^,^^5^ Excess visceral fat is a strong and independent predictor of insulin resistance, systemic inflammation, and cardiovascular risk, while glycated hemoglobin (HbA1c) remains the gold-standard marker of long-term glycemic control and a well-established predictor of micro- and macro-vascular complications.^4^^,^^6^^,^^7^ Therapeutic strategies capable of safely improving metabolic health independently of weight loss remain rare and mechanistically underexplored.^1^^,^^8^

Cannabinoid receptor type 1 (CB1R) peripheral signaling represents a compelling target for adipose tissue remodeling, despite the fact that previous centrally active CB1R compounds were clinically limited by neuropsychiatric adverse effects.^9^^,^^10^^,^^11^ Pep19 (DIIADDEPLT), an intracellular peptide,^12^^,^^13^^,^^14^^,^^15^^,^^16^ was functionally characterized as an inverse agonist of the CB1R using conformational specific antibodies.^17^^,^^18^^,^^19^ Pharmacological characterization in white adipose tissue and 3T3-L1 differentiated cells indicates that Pep19 activates pERK1/2 and AKT signaling, while upregulating uncoupling protein 1 (UCP1) expression; these effects were blocked by the CB1R antagonist AM251, suggesting that Pep19 may act as an unconventional cannabinoid receptor modulator, potentially engaging inverse agonist and/or biased agonist mechanisms.^20^ Consistent with these *in vitro* findings, oral Pep19 administration to two distinct rodent models of diet-induced obesity (Wistar rats and Swiss mice) promoted systemic improvements, including reductions in mesenteric (visceral) adipose tissue, triglycerides, cholesterol, and blood pressure, while attenuating liver inflammation and hepatic fat accumulation and reducing the absolute mass of the endocrine pancreas; it also stimulated adipose tissue browning and enhanced insulin sensitivity.^20^^,^^21^ Pep19 demonstrated an absence of neurotoxicity or central nervous system activity, as evidenced by the cannabinoid tetrad, a lack of brain c-fos induction, and the absence of depressive or anxious-like behaviors.^20^^,^^21^ Pep19 safety profile was reinforced in a 28-day study with healthy Beagle dogs, which showed a reduction in body weight without changes in caloric intake or biochemical parameters^22^; this safety profile of Pep19 overcomes the historical limitations of centrally acting CB1R compounds.^9^^,^^10^^,^^11^ Pep19 has been recognized as generally recognized as safe status, based on scientific procedures in accordance with 21 C.F.R. § 170.30 (a) and (b) and conforms with guidance § 170.36 from the United States of America Food and Drug Administration. Consistently, a previous 60-day triple-blind early-stage clinical trial in a US obese population showed that 5 mg Pep19 orally administered daily at bedtime, significantly reduces visceral fat while preserving lean mass while having no adverse effects.^23^

Here, we investigated whether Pep19 can induce metabolic improvement through remodeling of adipose tissue distribution despite stable body weight. To address this question, we conducted a randomized, double-blind, placebo-controlled clinical trial in adults with obesity, evaluating the effects of two Pep19 doses (5 mg and 10 mg) over a 90-day intervention period. Beyond confirming prior observations, this study was designed to examine whether Pep19 uncouples metabolic improvements from weight loss by integrating imaging-based assessment of regional fat distribution with longitudinal metabolic analyses. By combining clinical endpoints with mechanistic insight into adipose tissue biology, the present work aims to define a weight-independent metabolic modulation strategy, and to further characterize intracellular peptides as an emerging pharmacological concept targeting peripheral CB1R signaling.

## Results

### Study population and baseline characteristics

**Figure 1:**
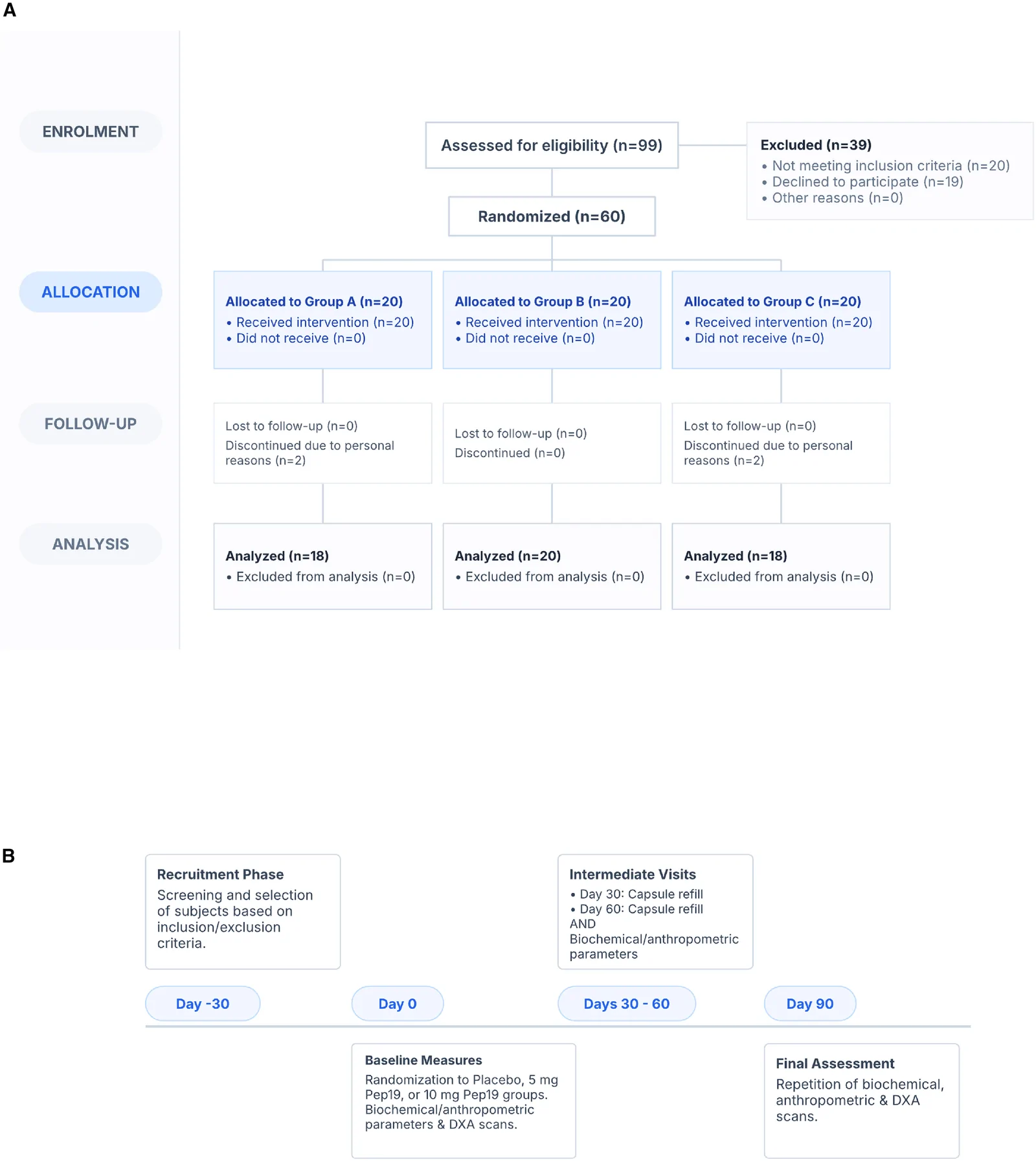
CONSORT trial flow diagram and experimental design of the Pep19 clinical trial (A) Study flowchart.^24^^,^^25^ CONSORT flowchart detailing participant screening (*n* = 99), exclusion (39), and randomization (60). Participants were allocated into three balanced arms: group A (Placebo), group B (low-dose Pep19), or group C (high-dose Pep19). Final analysis sets included 18 participants in group A (two lost to follow-up due to personal reasons), 20 in group B, and 18 in group C (two lost to follow-up due to personal reasons). (B) Study design. Experimental timeline outlining the chronological 90-day study protocol. The sequence tracks from the recruitment phase (day −30) through baseline measures and randomization (day 0), intermediate visits with capsule refills and monitoring (days 30 and 60), to final assessments (day 90). Abbreviations: CONSORT, consolidated standards of reporting trials; DXA, dual-energy X-ray absorptiometry.

A total of 99 individuals from the FMABC University Center (Santo André, SP, Brazil) were screened for eligibility. Of these, 39 were excluded (did not meet inclusion criteria or declined to participate). Sixty participants were enrolled and randomized to the intervention groups (BMI 30–39.9 kg/m^2^); participants were adults with obesity. Fifty-six participants completed the 90-day intervention and were included in the final analysis. Four participants discontinued the study for reasons unrelated to treatment or adverse events. Figure 1 illustrates the study flowchart and overall experimental design.^24^^,^^25^ Baseline demographic, anthropometric, metabolic, and safety parameters were comparable across the placebo, Pep19 5 mg, and Pep19 10 mg groups, with no statistically significant differences observed at study entry (Table 1). Participants in all groups exhibited obesity with largely preserved metabolic function, although several individuals presented borderline elevations in insulin resistance markers consistent with early metabolic dysfunction. Baseline inflammatory markers and safety-related laboratory parameters were within normal ranges and similarly distributed across groups, indicating successful randomization and an appropriate starting point for evaluation of treatment-related effects.

**Table 1:** Baseline demographic and characteristics of enrolled subjects in absolute values

Outcome	Placebo	5 mg	10 mg
Demographic data			
Age, years	41.00 [30.25–49.50]	38.00 [24.00–48.25]-	33.00 [29.00–41.00]
Sex			
Female	10 (56%)	12 (60%)	11 (61%)
Male	8 (44%)	8 (40%)	7 (39%)
Primary endpoint			
A/G ratio	1.00 [0.91–1.08]	0.98 [0.96–1.13]	1.04 [0.98–1.14]
Secondary endpoints			
Body weight (kg)	101.19 ± 14.15	101.38 ± 16.44	102.99 ± 12.70
BMI (kg/m^2^)	35.02 ± 3.46	35.58 ± 3.22	35.69 ± 2.31
Skinfolds sum (mm)	192.06 ± 25.00	202.39 ± 24.47	202.69 ± 21.08
Abdominal skinfold (mm)	35.44 ± 4.91	36.26 ± 4.33	37.20 ± 6.03
Hip circumference (cm)	118.29 ± 6.44	117.70 ± 6.62	117.28 ± 5.84
Waist circumference (cm)	99.31 ± 13.03	99.45 ± 14.54	100.94 ± 8.47
Abdominal circumference (cm)	108.53 ± 13.31	110.00 ± 13.49	110.13 ± 7.89
% of body fat	29.87 ± 5.16	31.02 ± 3.94	30.33 ± 4.96
Fat mass (kg)	29.87 ± 4.79	31.04 ± 5.44	30.82 ± 3.46
Lean mass (kg)	60.38 ± 8.15	69.27 ± 13.59	67.27 ± 9.88
Glucose (mg/dL)	91.95 [86.40–97.47]	88.00 [85.30–100.62]	89.70 [85.20–99.80]
Glycated hemoglobin HbA1c (%)	5.32 ± 0.38	5.37 ± 0.37	5.59 ± 0.51
Insulin (μU/mL)	14.70 [12.90–23.50]	16.15 [10.55–21.50]	21.80 [15.17–31.85]
HOMA-IR	3.37 [2.36–5.10]	3.01 [2.37–4.95]	4.12 [3.78–7.35]
HDL cholesterol (mg/dL)	45.05 ± 8.66	47.93 ± 7.64	43.89 ± 9.66
LDL cholesterol (mg/dL)	119.25 ± 31.87	114.17 ± 30.45	112.65 ± 32.90
Total cholesterol (mg/dL)	188.98 ± 41.37	184.38 ± 32.57	179.12 ± 35.73
Triglycerides (mg/dL)	102.30 [83.15–154.65]	99.95 [78.68–109.00]	94.65 [71.45–139.65]
VLDL cholesterol (mg/dL)	20.46 [16.62–30.93]	19.97 [15.73–21.81]	18.95 [14.27–27.93]
Interleukin-6 (pg/mL)	2.00 [2.00–3.03]	2.10 [2.00–2.80]	2.60 [2.00–2.80]
Tumor necrosis factor-alpha (pg/mL)	8.00 [7.08–9.00]	9.00 [7.75–10.40]	9.00 [8.00–10.30]
Neutrophil-to-lymphocyte ratio	1,779.10 [1,323.23–2,267.74]	1,742.56 [1,205.80–2,219.20]	1,687.27 [1,552.15–2,289.50]
C-reactive protein (mg/dL)	2.63 [2.02–3.83]	2.74 [1.58–4.58]	3.15 [2.46–6.09]
Alanine aminotransferase (U/L)	20.00 [14.50–31.75]	21.50 [15.75–30.75]	17.50 [12.50–40.50]
Aspartate aminotransferase (U/L)	18.50 [17.00–24.75]	18.00 [14.75–23.50]	21.00 [15.00–25.75]
Gamma glutamyl transferase (U/L)	29.00 [24.00–38.00]	29.50 [21.25–40.75]	25.00 [19.00–35.00]
Systolic blood pressure (mmHg)	124.00 [120.00–135.25]	124.50 [120.00–134.25]	124.00 [115.00–128.00]
Safety outcomes			
Urea (mg/dL)	29.04 ± 5.43	31.45 ± 9.40	26.67 ± 7.31
Creatinine (mg/dL)	0.80 [0.80–0.90]	0.80 [0.70–1.02]	0.80 [0.70–0.97]
Uric Acid (mg/dL)	5.60 [4.93–6.22]	5.50 [4.28–6.90]	6.35 [5.32–6.88]
Hematocrit (%)	41.95 [38.75–46.00]	41.35 [40.20–44.82]	40.30 [38.42–47.32]
Erythrocytes (million/mm^3^)	4.83 [4.58–5.37]	4.94 [4.64–5.16]	4.81 [4.59–5.46]
Hemoglobin (g/dL)	14.56 ± 1.22	14.46 ± 1.25	14.39 ± 1.72
Iron concentration	1.30 [1.20–1.37]	1.25 [1.20–1.40]	1.30 [1.20–1.40]
Lymphocytes (%)	3,237.22 ± 607.89	3,346.50 ± 911.55	3,162.78 ± 691.42
Monocytes (%)	685.00 [617.50–835.00]	745.00 [605.00–882.50]	705.00 [547.50–737.50]
Leukocytes (%)	7,255.56 ± 1,551.55	7,870.00 ± 3,011.22	8,597.22 ± 2,269.65
Basophil (%)	70.00 [60.00–100.00]	70.00 [47.50–85.00]	50.00 [42.50–67.50]
Eosinophils (%)	21.00 [16.25–30.75]	26.00 [16.00–33.25]	18.00 [13.00–31.00]
Segmented neutrophils (cell/μL)	57,150.00 ± 6,594.14	55,710.00 ± 10,179.49	58,644.44 ± 8,582.14
Mean corpuscular hemoglobin (pg)	29.78 ± 1.52	29.00 ± 1.45	29.04 ± 1.70
Mean corpuscular hemoglobin conc. MCHC (g/dL)	34.50 ± 1.15	34.10 ± 1.33	34.15 ± 1.51
Platelets (mm^3^)	269.39 ± 47.07	294.75 ± 56.87	287.24 ± 51.83
Mean platelet volume MPV (μm^3^)	10.44 ± 1.03	9.85 ± 0.85	10.33 ± 0.87
Red cell distribution width RDW (%)	12.65 [12.33–13.17]	12.80 [12.47–13.32]	13.00 [12.50–13.50]
Free thyroxine T4 (ng/dL)	1.22 [1.06–1.33]	1.27 [1.11–1.46]	1.19 [1.11–1.38]
Thyroid-stimulating hormone TSH (μUI/mL)	1.90 [1.31–2.36]	1.85 [1.39–2.17]	1.80 [1.49–2.77]
Other outcomes			
Sleep quality	3.00 [3.00–5.00]	4.00 [3.00–5.00]	3.00 [2.12–4.00]
Physical activity			
Does not practice	11 (33%)	14 (47%)	11 (42%)
Practices	7 (21%)	6 (20%)	7 (27%)

Baseline demographic, anthropometric, metabolic, and safety characteristics of participants enrolled in the randomized, double-blind, placebo-controlled clinical trial. Baseline characteristics of participants allocated to placebo, 5 mg Pep19, and 10 mg Pep19 treatment groups before intervention. Primary endpoints included body composition, measured by DXA and anthropometric parameters; secondary endpoints comprised metabolic related biomarkers and inflammatory markers; safety outcomes assessed clinical safety. Additional exploratory outcomes included self-reported sleep quality and physical activity status. Continuous variables are presented as mean ± standard deviation (SD) for normally distributed data or median (interquartile range) for non-normally distributed data. Categorical variables are expressed as number (%). No statistically significant differences were observed among groups at baseline, indicating successful randomization. Abbreviations: BMI, body mass index; A/G ratio, android-to-gynoid fat distribution; HbA1c, glycated hemoglobin; HOMA-IR, homeostatic model assessment of insulin resistance; HDL, high-density lipoprotein; LDL, low-density lipoprotein; VLDL, very-low-density lipoprotein; MCHC, mean corpuscular hemoglobin concentration; MPV, mean platelet volume; RDW, red cell distribution width; TSH, thyroid-stimulating hormone; T4, free thyroxine.

### Primary endpoint

**Figure 2:**
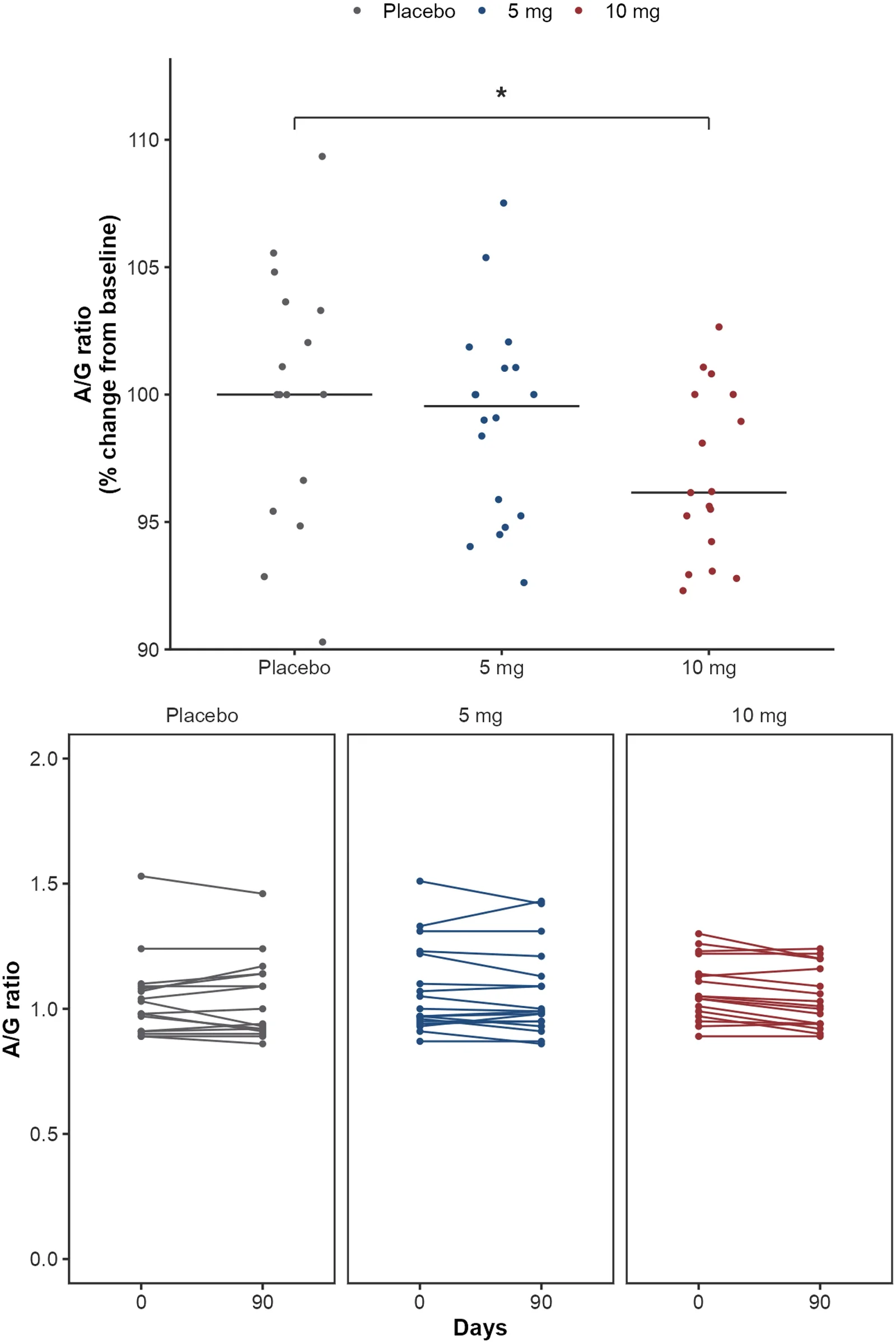
Primary efficacy endpoint: between-group difference in the change from baseline in the DXA-derived A/G ratio Top: individual participant data points for A/G ratio as a percentage of baseline at 90 days ∗*p* < 0.05 vs. placebo (ANCOVA). Bottom: individual participant trends for absolute A/G ratio values from day 0 to day 90. Note: Post-intervention imaging data were unavailable for three participants (placebo, *n* = 2; 5 mg Pep19, *n* = 1) due to scheduling and protocol timing constraints; horizontal bars indicate group median. Sample size: *n* = 16 (placebo), *n* = 19 (5 mg Pep19), and *n* = 18 (10 mg Pep19). Android-to-gynoid fat distribution: A/G ratio. 5 mg and 10 mg refer to the respective Pep19 treatment cohorts. The between-group difference (10 mg versus placebo) was statistically significant on the percentage-of-baseline scale (top; *p* = 0.037) but did not reach statistical significance on the absolute values (bottom; *p* = 0.058).

The prespecified primary efficacy endpoint was the between-group difference in the change from baseline to day 90 in the dual-energy X-ray absorptiometry (DXA)-derived android-to-gynoid fat distribution (A/G ratio). The results for the primary and secondary efficacy endpoints are summarized in Table 2, and longitudinal changes in the primary endpoint are shown in Figure 2. Compared with placebo, treatment with 10 mg Pep19 resulted in a greater reduction in A/G ratio from baseline. The observed mean between-group difference in percentage change from baseline was 3.19 percentage points (99.99% vs. 96.80%; Table 2). The prespecified mixed-effects model estimated an adjusted treatment effect of −3.58 percentage points (95% confidence interval [CI], −6.96 to −0.19; *p* = 0.037; Table 2). This finding was supported by the log/multiplicative analysis, which showed a 3.4% relative reduction (95% CI, −6.75% to −0.01%; *p* = 0.0495; Table 2). On the absolute scale, the adjusted between-group difference was −0.037 A/G ratio units (95% CI, −0.075 to +0.001; *p* = 0.058; Table 2). No evidence of a treatment effect on the A/G ratio was observed with 5 mg Pep19 (adjusted between-group difference, −1.16 percentage points; 95% CI, −4.44 to 2.12; *p* = 0.631; Table 2). Individual participant trajectories further showed that the reduction in A/G ratio among participants receiving 10 mg Pep19 was broadly consistent across individuals rather than being driven by a small number of extreme responders (Figure 2).

**Table 2:** Primary and secondary endpoints at the end of the study

Outcome	Placebo	5 mg	10 mg
Endpoints in absolute values at 90 days			
Primary endpoint			
A/G ratio	0.97 [0.92–1.14]	0.99 [0.95–1.12]	1.01 [0.94–1.16]
Secondary endpoints			
Body weight (kg)	101.08 ± 15.28	101.59 ± 16.69	103.59 ± 13.06
BMI (kg/m^2^)	34.94 ± 3.69	35.23 ± 3.27	35.91 ± 2.32
Skinfold sum (mm)	174.39 ± 22.94	175.07 ± 32.25	151.25 ± 49.46∗∗
Abdominal skinfold (mm)	34.94 ± 3.73	33.61 ± 4.64	31.57 ± 4.22∗∗
Hip circumference (cm)	117.88 ± 7.02	117.45 ± 6.79	117.78 ± 6.24
Waist circumference (cm)	99.94 ± 13.42	99.10 ± 15.08	100.44 ± 8.63
Abdominal circumference (cm)	109.93 ± 13.39	107.95 ± 14.90∗	109.20 ± 8.69
% of body fat	27.99 ± 4.86	28.82 ± 3.44	27.19 ± 3.71
Fat mass (kg)	28.02 ± 5.12	28.74 ± 4.90	28.35 ± 3.16
Lean mass (kg)	62.77 ± 9.08	72.53 ± 13.95	72.45 ± 7.87
Glucose (mg/dL)	98.19 ± 20.79	98.17 ± 12.74	93.38 ± 9.78
Glycated hemoglobin HbA1c (%)	5.38 ± 0.49	5.34 ± 0.35	5.22 ± 0.42∗
Insulin (μU/mL)	21.80 [12.85–26.45]	15.30 [9.10–22.70]	18.35 [15.35–19.70]
HOMA-IR	5.17 ± 3.38	3.21 ± 1.74∗	3.85 ± 1.60∗
HDL cholesterol (mg/dL)	47.40 ± 8.46	47.35 ± 7.28	44.79 ± 7.74
LDL cholesterol (mg/dL)	115.24 ± 33.61	113.67 ± 34.74	116.15 ± 39.35
Total cholesterol (mg/dL)	186.51 ± 41.05	179.21 ± 33.49	184.43 ± 41.36
Triglycerides (mg/dL)	96.80 [70.85–138.62]	94.70 [69.38–131.80]	113.50 [89.23–148.65]
VLDL cholesterol (mg/dL)	19.33 [14.17–27.72]	18.94 [13.88–26.56]	22.71 [17.80–29.73]
Interleukin-6 (pg/mL)	2.00 [2.00–2.10]	2.10 [2.00–2.35]	2.00 [2.00–2.25]
Tumor necrosis factor-alpha (pg/mL)	7.97 ± 2.65	8.80 ± 3.08	9.23 ± 4.12
Neutrophil to lymphocyte ratio	1762.55 ± 935.35	2054.64 ± 783.58	1771.78 ± 476.68
C-reactive protein (mg/dL)	2.12 [1.51–3.67]	2.51 [1.35–4.94]	2.53 [2.09–4.02]
Alanine aminotransferase (U/L)	18.00 [13.50–32.25]	22.00 [17.00–38.75]	18.00 [15.50–36.50]
Aspartate aminotransferase (U/L)	19.00 [16.25–22.00]	18.50 [17.00–26.75]	19.00 [16.25–24.50]
Gamma glutamyl transferase (U/L)	28.75 [22.05–41.50]	30.00 [19.25–40.00]	20.00 [17.50–32.50]
Systolic blood pressure (mmHg)	127.88 ± 11.54	128.50 ± 16.99	118.83 ± 10.35
Other outcomes			
Physical activity	7 (38.9%)	10 (50.0%)	5 (27.8%)
Sleep quality	4.00 [4.00–5.00]	4.25 [3.75–5.00]	4.00 [3.12–4.00]
Endpoints in % of baseline at 90 days			
Primary endpoint			
A/G ratio	99.99 ± 5.00	99.03 ± 3.98	96.80 ± 3.29∗
Secondary endpoints			
Body weight (kg)	99.82 ± 3.55	100.19 ± 2.26	100.57 ± 2.21
BMI	99.76 ± 3.58	99.86 ± 3.15	100.68 ± 2.48
Skinfold sum	91.06 ± 8.27	87.17 ± 14.39	74.15 ± 25.64∗∗∗
Abdominal skinfold	99.33 ± 8.42	92.90 ± 10.13	86.78 ± 14.43∗
Hip circumference	99.66 ± 2.94	99.79 ± 1.71	100.43 ± 1.82
Waist circumference	100.66 ± 3.58	99.62 ± 3.00	99.97 ± 2.98
Abdominal circumference	101.38 ± 3.81	98.01 ± 4.59∗	99.13 ± 2.79
% of body fat	93.79 ± 5.80	94.35 ± 3.78	91.01 ± 11.05
Fat mass	93.68 ± 7.33	94.85 ± 4.78	91.72 ± 12.58
Lean mass	103.99 ± 5.38	104.81 ± 3.03	108.21 ± 5.47
Glucose	106.07 ± 11.58	105.99 ± 19.31	101.49 ± 13.19
Glycated hemoglobin HbA1c (%)	100.84 ± 3.93	97.88 ± 5.84	93.96 ± 5.06∗
Insulin	126.03 ± 68.13	110.74 ± 59.00	96.78 ± 32.11
HOMA-IR	142.10 ± 87.72	87.18 ± 39.21∗	72.98 ± 15.45∗
HDL cholesterol	106.45 ± 14.83	99.64 ± 12.70	103.92 ± 15.46
LDL cholesterol	97.60 ± 16.35	99.06 ± 26.03	101.89 ± 23.36
Total cholesterol	99.55 ± 12.65	97.61 ± 12.84	103.14 ± 11.34
Triglycerides	100.47 ± 27.35	103.74 ± 33.74	114.35 ± 53.96
VLDL cholesterol	100.42 ± 27.36	103.96 ± 34.01	114.43 ± 54.24
Interleukin-6 (pg/mL)	100.00 [75.00–100.00]	100.00 [87.53–107.50]	92.31 [76.32–100.00]
Tumor necrosis factor-alpha (pg/mL)	106.82 ± 27.46	97.66 ± 46.50	116.59 ± 74.79
Neutrophil to lymphocyte ratio	90.98 [72.67–106.69]	111.66 [96.13–126.53]	100.86 [87.70–109.34]
C-reactive protein	88.71 [62.42–117.99]	102.60 [89.46–130.45]	86.40 [68.50–120.51]
Alanine aminotransferase (U/L)	100.00 [86.58–114.66]	104.58 [89.86–134.38]	100.00 [82.42–127.68]
Aspartate aminotransferase (U/L)	97.73 ± 22.11	107.88 ± 33.02	102.54 ± 21.94
Gamma glutamyl transferase (U/L)	98.60 [88.68–115.31]	96.57 [81.25–120.98]	95.24 [80.00–103.70]
Systolic blood pressure (mmHg)	99.47 ± 8.77	101.26 ± 8.38	97.66 ± 8.12
Atherogenic coefficient	92.77 ± 16.27	98.31 ± 16.44	102.68 ± 22.89
QUICKI	99.86 ± 7.07	99.30 ± 7.81	97.26 ± 5.90
HOMA-B	117.59 ± 54.86	112.48 ± 54.47	98.26 ± 23.54
Other outcomes			
Sleep quality	133.33 [100.00–166.67]	100.00 [80.00–110.71]∗	100.00 [100.00–145.83]

Effects of Pep19 treatment on primary and secondary endpoints after intervention. Absolute end-of-study values and percentage of baseline are shown for participants receiving placebo, Pep19 5 mg, or Pep19 10 mg. Primary endpoints included body composition measured by anthropometric parameters and DXA. Secondary endpoints comprised metabolic-associated biomarkers, including glycemic control, insulin sensitivity, lipid profile, inflammatory markers, and blood pressure. Additional exploratory outcomes included sleep quality and physical activity. Continuous variables are presented as mean ± standard deviation (SD) for normally distributed data or median (interquartile range) for non-normally distributed data. Percentage of baseline was calculated for each participant and summarized by treatment group. Statistical significance denotes between-group differences versus placebo, adjusted for multiple comparisons using the Dunnett method (many-to-one). For the primary endpoint (A/G ratio, percentage of baseline), the observed mean values at day 90 were 99.99% in the placebo group and 96.80% in the 10 mg Pep19 group, corresponding to a raw between-group difference of −3.19 percentage points. The prespecified mixed-effects model estimated an adjusted treatment effect of −3.58 percentage points (95% confidence interval [CI], −6.96 to −0.19; *p* = 0.037). Supporting analyses yielded a 3.4% relative reduction on the log/multiplicative scale (95% CI, −6.75% to −0.01%; *p* = 0.0495) and an adjusted between-group difference of −0.037 A/G ratio units on the absolute scale (95% CI, −0.075 to +0.001; *p* = 0.058). ∗*p* < 0.05, ∗∗*p* < 0.01, ∗∗∗*p* < 0.001, ANCOVA. Abbreviations: A/G ratio, android-to-gynoid fat distribution; BMI, body mass index; HbA1c, glycated hemoglobin; HOMA-IR, homeostatic model assessment of insulin resistance; QUICKI, quantitative insulin sensitivity check index; HDL, high-density lipoprotein; LDL, low-density lipoprotein; VLDL, very-low-density lipoprotein; HOMA-B, homeostatic model assessment of beta-cell function.

### Secondary endpoints

Secondary endpoints included anthropometric measures (body weight, BMI, waist and hip circumferences, skinfold thicknesses, total fat mass, lean body mass, and other DXA-derived body composition variables), metabolic parameters with a primary focus on insulin-glucose homeostasis, hemodynamic variables, inflammatory biomarkers, and lipid profile.

#### Body composition

Consistent with the primary endpoint, Pep19 treatment produced selective improvements in regional adiposity without significant changes in overall body weight or global body composition (Table 2). Participants receiving 10 mg Pep19 exhibited significant reductions in total skinfold thickness (*p* < 0.01) and abdominal skinfold thickness (*p* < 0.05), whereas participants receiving 5 mg Pep19 showed a significant reduction in abdominal circumference relative to baseline compared with placebo (*p* < 0.05). Body weight, BMI, total fat mass, percentage body fat, and lean body mass remained unchanged across treatment groups (Table 2).

#### Glucose homeostasis

**Figure 3:**
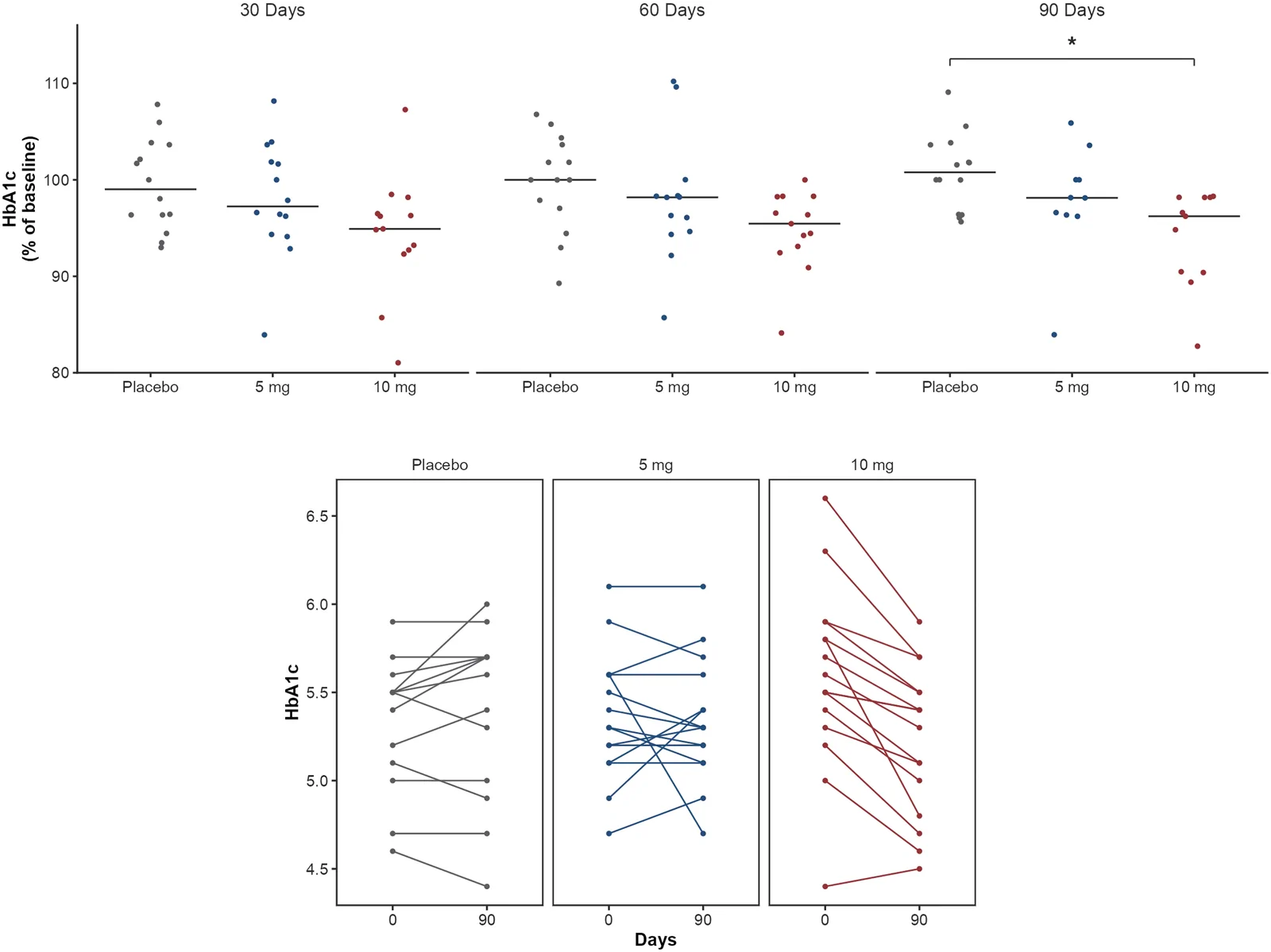
Effects of placebo and Pep19 on HbA1c levels Top: individual HbA1c responses across treatment groups over time. Scatterplots showing HbA1c values expressed as a percentage of baseline (100% = no change from day 0) for placebo (gray), 5 mg Pep19 (blue), and 10 mg Pep19 (red) groups at day 30, day 60, and day 90. Each point represents an individual participant; horizontal bars indicate group means. A dose-dependent reduction in HbA1c was observed across all timepoints, with the 10 mg Pep19 group reaching statistical significance versus placebo at day 90 (∗*p* < 0.05 vs. Placebo - ANCOVA). Sample size: *n* = 16 (placebo); *n* = 20 (5 mg Pep19); *n* = 17 (10 mg Pep19). Bottom: individual participant trends for absolute HbA1c values from day 0 to day 90.

**Figure 4:**
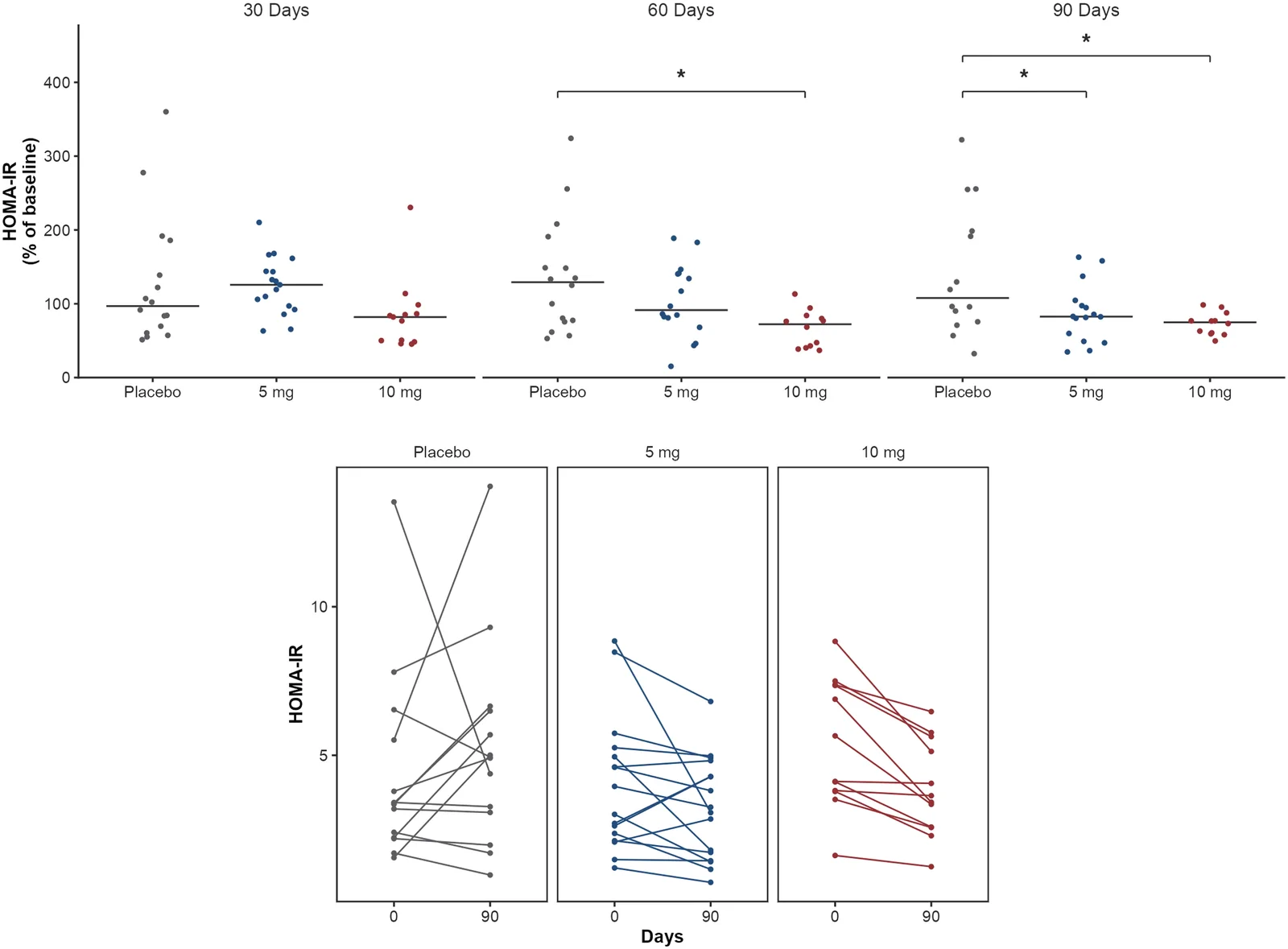
Effects of Pep19 on insulin resistance (HOMA-IR) Top: scatterplots showing HOMA-IR values expressed as a percentage of baseline (100% = no change from day 0) for placebo (gray), 5 mg Pep19 (blue), and 10 mg Pep19 (red) groups at day 30, day 60, and day 90. Each point represents an individual participant; horizontal bars indicate group means. The 10 mg Pep19 group reached statistical significance versus placebo at day 60 and day 90 (∗*p* < 0.05 - ANCOVA), while the 5 mg Pep19 group reached significance only at day 90 (∗*p* < 0.05 - ANCOVA). Sample size: *n* = 16 (placebo); *n* = 20 (5 mg Pep19); *n* = 17 (10 mg Pep19). Bottom: individual participant trends for absolute HOMA-IR values from day 0 to day 90.

Pep19 treatment was associated with improvements in selected measures of glucose homeostasis and insulin sensitivity (Table 2 and Figures 3 and 4). Participants receiving 10 mg Pep19 showed a significant reduction in HbA1c compared with placebo after 90 days despite stable fasting glucose concentrations (Table 2 and Figure 3). Participants receiving 5 mg Pep19 exhibited significant reductions in HOMA-IR at day 90, whereas significant reductions were observed as early as day 60 in the 10 mg Pep19 group and were maintained through day 90 (Figure 4). Insulin concentrations also showed a downward trend in both Pep19-treated groups (Table 2).

#### Hemodynamics

Hemodynamic parameters remained stable throughout the intervention (Table 2). Although systolic blood pressure tended to decrease in the 10 mg Pep19 group, the between-group difference did not reach statistical significance.

#### Inflammatory markers and clinical biochemistry

Liver enzymes, including alanine aminotransferase, aspartate aminotransferase, and gamma-glutamyl transferase, remained within the normal range throughout the study (Table 2). Likewise, inflammatory biomarkers, including C-reactive protein, interleukin-6 (IL-6), tumor necrosis factor-α (TNF-α), and neutrophil-to-lymphocyte ratio (NLR), as well as lipid profile parameters (total cholesterol, high-density lipoprotein [HDL]; low-density lipoprotein [LDL]; very-low-density lipoprotein [VLDL], and triglycerides), did not differ significantly among treatment groups (Table 2).

### Exploratory endpoints

Exploratory analyses included physical activity (Tables 1 and 2), dietary intake (Figure S1), and sleep quality (Table 2). Dietary intake and physical activity remained stable throughout the intervention, suggesting that the observed changes in body fat distribution and metabolic parameters were unlikely to be attributable to behavioral modifications. Self-reported sleep quality showed a nominally significant difference between the 5 mg Pep19 group and placebo on the percentage-of-baseline scale (Table 2); however, this exploratory finding was not robust to log-transformation, was not observed with the 10 mg dose, and should be interpreted with caution.

### Cluster analysis

**Figure 5:**
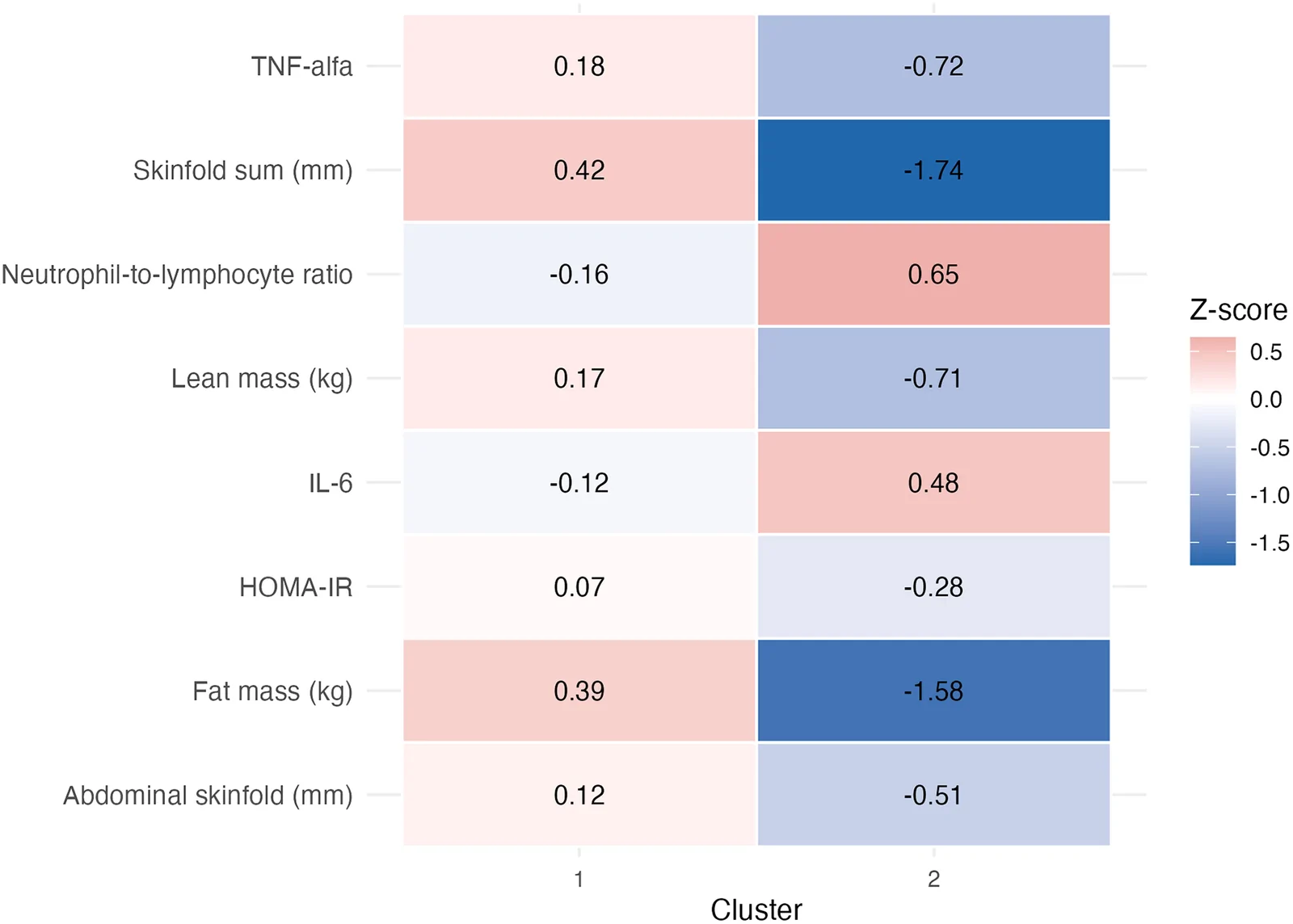
Standardized centroid profiles of k-means response clusters at day 90 Heatmap of cluster centroids (*Z* scores) from k-means clustering of day 90 outcomes expressed as a percentage of each participant’s baseline. Rows correspond to the clustering features: abdominal skinfold thickness, total fat mass (kg), HOMA-IR, IL-6, lean mass (kg), neutrophil-to-lymphocyte ratio (NLR), sum of skinfolds (mm), and TNF-α. Columns represent the two clusters (cluster 1, *n* = 45; cluster 2, *n* = 11: 6 participants from the 5 mg Pep19 group and 5 from the 10 mg Pep19 group). Red shading indicates centroid values above the cohort mean and blue shading indicates values below the cohort mean. Cluster 2 is characterized by pronounced reductions in adiposity-related features (fat mass, sum of skinfolds, abdominal skinfold) and is exclusively composed of Pep19-treated participants, identifying it as the high-response phenotype.

To investigate whether the metabolic and body composition effects of Pep19 reflected a uniform response or the presence of distinct response phenotypes, we performed an unsupervised k-means clustering analysis on day 90 values expressed as a percentage of each participant’s baseline. Two clusters with markedly different response profiles were identified (Figure 5). Cluster 1 (*n* = 45) included the majority of participants and showed minimal change from baseline across all features (centroid z-scores between −0.16 and +0.42). In contrast, cluster 2 (*n* = 11) represented a high-response phenotype, characterized by pronounced reductions in adiposity-related measures [total fat mass (*Z* = −1.58), sum of skinfolds (*Z* = −1.74), abdominal skinfold (*Z* = −0.51), and lean mass (*Z* = −0.71)] together with a decrease in HOMA-IR (*Z* = −0.28) and a substantial reduction in TNF-α (*Z* = −0.72). Importantly, cluster 2 was composed exclusively of Pep19-treated participants (6 from the 5 mg group, and 5 from the 10 mg group), with no placebo-treated participants meeting this response profile. The elevated IL-6 (*Z* = +0.48) and neutrophil-to-lymphocyte ratio (*Z* = +0.65) centroids in cluster 2 likely reflect the intrinsic variability of these markers and were not accompanied by concordant changes in other inflammatory parameters. These findings indicate that, although mean changes in the overall cohort were modest, a defined subgroup of Pep19-treated participants experienced a coordinated reduction in adiposity and improvement in insulin resistance and TNF-α, supporting the existence of a responder phenotype to Pep19 treatment.

### Safety

Safety was assessed through renal, hepatic, hematological, and endocrine parameters. No treatment-related toxicological signals or clinically relevant laboratory abnormalities were observed throughout the intervention (Table S1), supporting the favorable short-term safety and tolerability profile of Pep19.

## Discussion

The present study provides evidence supporting a favorable effect of Pep19 on adipose tissue distribution, as reflected by a significant reduction in the A/G ratio in the prespecified primary analysis. Supporting analyses yielded estimates of similar magnitude and direction, although the absolute-scale analysis did not reach the conventional threshold for statistical significance. Together, these findings demonstrate consistency across analytical approaches while indicating greater uncertainty around the absolute-scale estimate. The absence of significant changes in body weight or total fat mass suggests that improving regional adipose tissue distribution may contribute to metabolic benefits without requiring substantial weight loss. Improvements in selected metabolic parameters, including insulin resistance and HbA1c, are consistent with this hypothesis but should be interpreted cautiously because these were secondary outcomes. Although these findings are compatible with the biological activity previously reported for Pep19 in preclinical studies, the present clinical trial did not evaluate target engagement or mechanistic biomarkers; therefore, the underlying biological mechanisms remain to be established.

In addition to the primary endpoint, treatment with 10 mg Pep19 was associated with a sustained reduction in HbA1c accompanied by a decline in HOMA-IR, despite stable fasting glucose concentrations, findings that are consistent with improved insulin sensitivity. Because these were secondary outcomes, they should be interpreted as exploratory. Unlike traditional antidiabetic agents typically rely on insulin secretagogues,^26^ renal glucose excretion (SGLT2i),^27^^,^^28^ or appetite suppression,^29^^,^^30^^,^^31^ Pep19 exhibited a safe and distinct metabolic profile, without effects on body weight, BMI or lean mass. The reduction in HbA1c despite unchanged fasting glucose and food intake changes points to improvements in integrated or postprandial glycemic control rather than acute fasting effects, potentially reflecting enhanced metabolic efficiency at the peripheral level. This distinction is clinically relevant, as HbA1c integrates cumulative glucose exposure and is more closely linked to long-term cardiometabolic risk than isolated fasting glucose values.^7^^,^^32^^,^^33^^,^^34^ The selective reduction in HbA1c may reflect improved integrated glycemic control not captured by fasting biomarkers alone.

Recent evidence helps reconcile previous discrepancies^35^ in hepatic CB1R biology by demonstrating that receptor function is highly dependent on metabolic state; whereas CB1R promotes Gi/o-mediated lipolysis under lean conditions, obesity induces a pathological shift toward Gs-mediated lipogenesis, driven in part by GPR3 and oleic acid priming.^36^ Although target engagement and downstream CB1R signaling were not assessed in the present clinical trial, these findings provide a plausible framework for interpreting the metabolic effects observed with Pep19. Based on its previously reported atypical pharmacological profile at CB1R,^21^ one hypothesis is that Pep19 acts as a context-dependent functional modulator, potentially favoring a lean-like signaling state characterized by enhanced Gi/o-AMPK-associated fatty acid oxidation and reduced Gs-SREBP-1c-mediated lipogenesis. Such a mechanism could contribute to the reductions in central adiposity and improvements in insulin sensitivity observed in this study; however, this hypothesis remains to be directly tested in future mechanistic studies.

Regional adipose depots exert distinct metabolic effects, with visceral fat contributing disproportionately to insulin resistance and cardiometabolic risk.^2^^,^^3^^,^^6^^,^^37^ The observed shift toward a more favorable fat distribution pattern suggests redistribution of lipid storage away from metabolically deleterious compartments. Although hepatic fat content was not directly measured herein, the reduction in regional adipose distribution is consistent with decreased portal free fatty acid flux and attenuation of hepatic lipotoxicity. By targeting the visceral-hepatic axis directly, Pep19 may mitigate key drivers of hepatic insulin resistance and hyperinsulinemia while avoiding the central adverse effects associated with first-generation cannabinoid therapies.^11^^,^^31^^,^^38^ Because previous preclinical^20^^,^^21^ and clinical^23^ studies suggested that Pep19 may preferentially affect regional adipose tissue distribution rather than total adiposity; therefore, the change from baseline to day 90 in the A/G ratio was selected as the prespecified primary endpoint. The 10 mg Pep19 dose was associated with a consistent pattern of changes across adiposity and metabolic outcomes, including reductions in the A/G ratio, abdominal skinfold thickness, skinfold sum, HbA1c, and HOMA-IR. Although the present study was not powered to formally compare doses, this pattern may suggest differential engagement of metabolic pathways depending on treatment dose of Pep19. Lower doses may preferentially influence early insulin signaling dynamics, whereas higher doses may promote more substantial structural remodeling of adipose tissue compartments. Such dose-dependent functional divergence warrants further investigation and may prove relevant for optimizing therapeutic strategies according to specific metabolic objectives.

Unsupervised cluster analysis revealed that the metabolic effects of Pep19 may be more robust than suggested by conventional group mean comparisons. A distinct high-response cluster was identified exclusively among Pep19-treated participants, with no placebo-treated individuals exhibiting the same response profile. This subgroup demonstrated a coordinated pattern of metabolic improvement characterized by marked reductions in abdominal skinfold thickness, total fat mass, and overall skinfold sum, accompanied by decreases in HOMA-IR and TNF-α, despite the absence of significant body weight reduction. It is worth noting that, unlike TNF-α, IL-6 and C-reactive protein levels were below normal baseline values in all subjects. These findings suggest that Pep19 may preferentially benefit a metabolically responsive subset of individuals, consistent with the existence of a responder phenotype. Such heterogeneity is highly relevant in obesity therapeutics, where biologically meaningful responses are often obscured by interindividual variability when only aggregate treatment effects are considered. The identification of a Pep19-responsive subgroup reinforces the concept that peripheral CB1R modulation can induce targeted adipose remodeling and coordinated metabolic improvement without generalized weight reduction, while also supporting a precision-medicine framework in which future therapeutic use may be optimized through biomarker-guided patient stratification.

Whereas an earlier clinical study in an American cohort showed that Pep19 reduced body weight by only 2.0% on average, it produced a substantially greater reduction in visceral adipose tissue, with a mean decrease of approximately 17%.^23^ Together with the present findings demonstrating improvements in body fat distribution without significant weight loss, these results suggest that Pep19 primarily targets adipose tissue remodeling rather than body mass *per se*. This discrepancy may reflect differences in cohort characteristics, including age distribution, BMI range, genetic background (e.g., *CNR1* polymorphisms), and dietary patterns, all of which may influence endocannabinoid tone and metabolic responsiveness. The persistence of favorable metabolic effects despite unchanged body mass further supports the notion that Pep19 acts through mechanisms that are at least partially independent of weight loss, reinforcing its distinct pharmacological profile.

Pep19-induced metabolic improvements occurred without measurable loss of lean mass, suggesting that the observed changes were not driven by generalized catabolism or nonspecific weight reduction. Preservation of lean tissue is particularly relevant in obesity therapeutics, as reductions in skeletal muscle mass can compromise long-term metabolic health and functional capacity. The apparent ability of Pep19 to promote selective adipose remodeling while maintaining lean body composition may therefore represent a clinically advantageous profile compared with interventions that improve metabolic outcomes primarily through indiscriminate tissue loss.^39^^,^^40^^,^^41^ The absence of significant changes in circulating lipid parameters may reflect the relatively short intervention period and the predominantly normolipidemic baseline status of the cohort, suggesting that adipose tissue remodeling and insulin sensitization may precede detectable alterations in systemic lipid homeostasis. Moreover, the absence of elevated baseline pro-inflammatory markers, such as IL-6 and C-reactive protein, may have limited our ability to detect the anti-inflammatory effects of Pep19, aside from those revealed by unsupervised cluster analysis.^7^^,^^42^^,^^43^^,^^44^ Furthermore, systemic inflammation is often more strongly associated with total adiposity than with regional fat redistribution. Because Pep19 selectively altered adipose tissue distribution without substantially reducing total fat mass, detectable changes in circulating inflammatory markers were not expected over this relatively short, 90-day intervention.

Reductions in metabolically deleterious fat depots, HbA1c, and HOMA-IR, even within the high-normal or borderline range, may be clinically meaningful, as they can reduce cumulative metabolic stress and potentially delay progression toward overt metabolic dysfunction.^45^^,^^46^ The present study was designed to evaluate Pep19 in a pre-pathological population, with the broader therapeutic goal of preventing the transition from metabolic risk to established disease while avoiding the adverse effects commonly associated with existing pharmacological interventions. Demonstrating a shift toward the lower, healthier end of the normal range is therefore consistent with the intended mechanism of Pep19 to improve metabolic efficiency before irreversible tissue damage occurs. Because an HbA1c of approximately 5.7% represents the lower threshold for prediabetes, conventional pharmacological interventions are generally not indicated in otherwise healthy individuals at this stage, limiting opportunities for early and safe intervention. In this context, Pep19 that was well tolerated and without reported adverse effects in both the present and previous studies,^22^^,^^23^ may represent a promising option for individuals with normal or borderline-elevated HbA1c and HOMA-IR who nevertheless remain at increased long-term metabolic risk.

Altogether, these findings support further clinical investigation of Pep19. Larger and longer-term studies are needed to confirm the observed changes in regional adipose tissue distribution and selected metabolic parameters, and to determine whether these findings translate into meaningful long-term cardiometabolic benefits.

### Limitations of the study

Certain limitations should be acknowledged. Although the 90-day follow-up allowed assessment of the prespecified primary endpoint and selected secondary outcomes, longer-term studies are needed to determine the durability and clinical relevance of these findings. The relatively modest sample size may have limited the ability to detect differences in some secondary endpoints, particularly inflammatory biomarkers, and these secondary analyses should therefore be considered exploratory. Although participants were instructed to maintain their usual diet and physical activity, these behaviors were not objectively monitored and may have contributed to residual variability; therefore, the stability of these variables supports, but does not establish, weight-independent metabolic effects, and causal interpretations need to be avoided. In addition, baseline insulin resistance indices were numerically higher in the 10 mg Pep19 group, which may have influenced the magnitude of the observed changes in HOMA-IR. Thus, the present study provides evidence supporting improved metabolic regulation rather than definitive proof of improved insulin sensitivity. The study was not designed to investigate the molecular mechanisms underlying Pep19 action. The absence of adipokine profiling, target-engagement assessments, or other mechanistic biomarkers precludes direct evaluation of the biological pathways responsible for the observed clinical effects. Consequently, any mechanistic interpretation remains speculative and should be viewed in the context of previous preclinical studies rather than as evidence generated by the present clinical trial. The exploratory cluster analysis and the inclusion of participants from a single geographic setting require confirmation in larger, independent, and more diverse populations before the identified responder profile and the broader clinical applicability of these findings can be established.

## Resource availability

### Lead contact

Requests for further information and resources should be directed to and will be fulfilled by the lead contact, Fernando L.A. Fonseca (profferfonseca@gmail.com).

### Materials availability

This study did not generate new unique reagents.

### Data and code availability

• All anonymized raw data are available at supplemental information.
• This paper does not report original code. R script is available at supplemental information.
• Any additional information required to reanalyze the data reported in this work paper is available from the lead contact upon request.

## Acknowledgments

The authors are deeply grateful to Professor Itamar Raz, MD, PhD., Head of the Israeli National Council of Diabetes and Professor Lakshmi A Devi, Director of the Interdisciplinary Training in Drug Abuse Research Program at Icahn School of Medicine at Mount Sinai, for their critical review of the manuscript and valuable suggestions. Authors also deeply acknowledge Nimrod Elmish, Chief Executive Officer of Proteimax Biotechnology Israel, for his unwavering support, insightful feedback, and invaluable contributions, which have greatly enriched the present study. E.S.F. acknowledges the 10.13039/501100001807São Paulo Research Foundation (10.13039/501100001807FAPESP, grant 2016/04000-3) and the Brazilian National Research Council (CNPq; grant PQ 303096/2021-7). We deeply acknowledge the Fundação do ABC, Avenida Príncipe de Gales, 667, Bairro Príncipe de Gales, Santo André, SP, CEP 09060-590, Brazil, which funded all study procedures except for Pep19 capsule manufacturing and imaging assessments.

## Author contributions

D.V., J.R.d.S., R.O.S.S., S.H., F.L.F., R.R., R.A.R., L.F.M., E.S.F., A.S.H., and F.L.A.F. designed the study, analyzed the data, assisted in the experimental design, executed the experiments, and analyzed the data. D.V., J.R.d.S., R.O.S.S., S.H., F.L.F., R.R., R.A.R., A.K., L.F.M., E.S.F., A.S.H., and F.L.A.F. helped in the interpretation and discussed the results, commented, write and reviewed the manuscript.

## Declaration of interests

A.S.H., E.S.F., and A.K. are co-founders of Proteimax Biotechnology Israel.

## Declaration of generative AI and AI-assisted technologies in the writing process

During the preparation of this manuscript, the authors used artificial intelligence (AI) tools (ChatGPT, OpenAI; Gemini, Google) to assist with language editing and improvement of clarity. The AI tools were used exclusively for editorial support, including refinement of grammar, wording, and readability. All scientific content, interpretations, and conclusions were developed, reviewed, and approved by the authors. The authors take full responsibility for the integrity and accuracy of the manuscript.

## STAR★Methods

### Key resources table

**Table:** 

REAGENT or RESOURCE	SOURCE	IDENTIFIER
**Chemicals, peptides, and recombinant proteins**		

Pep19 (DIIADDEPLT; CAS 1536481–46–5)	Proteimax Biotechnology Israel Ltd	Proteimax Biotechnology Israel Ltd, Pardes Hana, Israel
Placebo, 5 mg Pep19 and 10 mg Pep19 capsules	Vegetable capsules (size 2) containing microcrystalline cellulose as the only excipient	Formularium Manipulation Pharmacy (São Paulo, Brazil)

**Critical commercial assays**		

Glucose	Roche Cobas 8000® modular analyzer series	Roche Diagnostics GmbH, Mannheim, Germany
Glycated hemoglobin (HbA1c)	Low-pressure liquid chromatography (LPLC)	Bio-Rad® system, Hercules, CA, USA
Insulin	Roche Cobas 8000® modular analyzer series	Roche Diagnostics GmbH, Mannheim, Germany
Alanine aminotransferase	Roche Cobas 8000® modular analyzer series	Roche Diagnostics GmbH, Mannheim, Germany
Aspartate aminotransferase	Roche Cobas 8000® modular analyzer series	Roche Diagnostics GmbH, Mannheim, Germany
Gamma glutamyl transferase	Roche Cobas 8000® modular analyzer series	Roche Diagnostics GmbH, Mannheim, Germany
IL6	Roche Cobas 8000® modular analyzer series	Roche Diagnostics GmbH, Mannheim, Germany
TNF-alfa	Roche Cobas 8000® modular analyzer series	Roche Diagnostics GmbH, Mannheim, Germany
C-reactive protein	Roche Cobas 8000® modular analyzer series	Roche Diagnostics GmbH, Mannheim, Germany
HDL Cholesterol	Roche Cobas 8000® modular analyzer series	Roche Diagnostics GmbH, Mannheim, Germany
LDL Cholesterol	Roche Cobas 8000® modular analyzer series	Roche Diagnostics GmbH, Mannheim, Germany
Total Cholesterol	Roche Cobas 8000® modular analyzer series	Roche Diagnostics GmbH, Mannheim, Germany
VLDL Cholesterol	Roche Cobas 8000® modular analyzer series	Roche Diagnostics GmbH, Mannheim, Germany
Triglycerides	Roche Cobas 8000® modular analyzer series	Roche Diagnostics GmbH, Mannheim, Germany
Creatinine	Roche Cobas 8000® modular analyzer series	Roche Diagnostics GmbH, Mannheim, Germany
Urea	Roche Cobas 8000® modular analyzer series	Roche Diagnostics GmbH, Mannheim, Germany
Uric Acid	Roche Cobas 8000® modular analyzer series	Roche Diagnostics GmbH, Mannheim, Germany
Thyroid-stimulating hormone	Roche Cobas 8000® modular analyzer series	Roche Diagnostics GmbH, Mannheim, Germany
Free thyroxine	Roche Cobas 8000® modular analyzer series	Roche Diagnostics GmbH, Mannheim, Germany
Complete blood count (CBC)	Roche Cobas 8000® modular analyzer series	Roche Diagnostics GmbH, Mannheim, Germany
Body composition	Dual-energy X-ray absorptiometry (DXA) using GE Healthcare Prodigy - Lunar DXA equipment	GE Healthcare, Madison, WI, USA

**Deposited data**		

Raw	This paper	dataset_pep19_deidentified.csv
Analyzed data	This paper	LMM_Results_Pep19_v5_dunnett.xlsx

**Software and algorithms**		

Statistical analyses	R (v4.5.1), with the *lme4* (v1.1-38) and *lmerTest* (v3.2-1) packages.	R Core Team, 2025
R script	This paper	LMM_analysis-vf-V5-dunnett.R
Anatomical segmentation and quantitative image processing	enCORE™	GE Healthcare, Madison, WI, USA
Subjects’ randomization	GraphPad Prism v10	GraphPad Software, Boston, Massachusetts USA

### Experimental model and study participant details

The randomized, double-blind, placebo-controlled trial was approved by the Ethics Committee of Centro Universitário FMABC (Santo André, SP, Brazil; CAAE: 84285324.20000.0082) and registered at the Brazilian Registry of Clinical Trials (UTN: U1111-1334-7473; RBR-10zchcdk). All participants provided written informed consent. Eligible participants were generally healthy adults (21–60 years) with BMI 30–39.9 kg/m^2^, fasting glucose <125 mg/dL, and HbA1c <6.5%. Exclusion criteria included pregnancy, lactation, and use of anti-obesity or antidiabetic medications or supplements.

Ninety-nine individuals were screened at the FMABC Endocrinology Outpatient Clinic; 39 were excluded (ineligible, *n* = 20; declined, *n* = 19). Sixty participants (23 males, 37 females) were randomized (1:1:1), stratified by sex and BMI, to receive placebo (*n* = 20), Pep19 5 mg (*n* = 20), or Pep19 10 mg (*n* = 20). Four participants dropout during the follow-up phase due to personal reasons (placebo, *n* = 2; Pep19 10 mg, *n* = 2), leaving 56 for analysis (placebo, *n* = 18; Pep19 5 mg, *n* = 20; Pep19 10 mg, *n* = 18).

### Method details

Sample size was calculated assuming a two-sided α of 0.05 and 80% power, yielding a minimum of 14 participants. Randomization was performed by an independent researcher using GraphPad Prism (v10), in a 1:1:1 ratio stratified by sex and BMI to homogenize the sample. Participants were instructed to self-administered one capsule daily at bedtime for 90 days. Blinding and allocation concealment were maintained until study completion and all data collected and processed into the analysis’s databank. Adherence was assessed by capsule counts every month.

Four participants did not complete the dual-energy X-ray absorptiometry (DXA) assessment at 90 days (placebo, *n* = 2; Pep19 5 mg, *n* = 1; Pep19 10 mg, *n* = 1), also due to personal reasons, and were excluded from DXA analyses. CONSORT trial flow diagram and experimental design of the Pep19 clinical trial were presented at Figure 1. 99 participants were screened, 39 were excluded, and 60 were randomized. Participants were allocated into three balanced arms: Group A (Placebo), Group B (low-dose Pep19), or Group C (high-dose Pep19). Final analysis sets included 18 participants in Group A (two lost to follow-up due to personal reasons), 20 in Group B, and 18 in Group C (two lost to follow-up due to personal reasons). Study design is showed in Figure 1B: Experimental timeline outlining the chronological 90-day study protocol. The sequence tracks from the recruitment phase (day −30) through baseline measures and randomization (day 0), intermediate visits with capsule refills and monitoring (days 30 and 60), to final assessments (day 90). The trial registry provides public access to the protocol and statistical analysis plan (https://ensaiosclinicos.gov.br/rg/RBR-10zchcdk). All anonymized raw data are available at Supplemental Material. Also, R script is available at supplemental information.

### Quantification and statistical analysis

#### Food inventory

Dietary intake was assessed using a 24-h dietary recall, a validated method in which participants report all foods and beverages consumed during the preceding 24-h period. This method provides an estimate of daily energy and nutrient intake and is commonly used in clinical and nutritional research^47^^,^^48^^,^^49^; given the inherent limitations of self-reported dietary assessment, the recall was used as a monitoring tool to identify substantial changes in dietary patterns that could confuse interpretation of metabolic outcomes. Physical activity was assessed using a World Health Organization Global Physical Activity Questionnaire-based binary classification, with participants categorized as sedentary (0) or physically active (1), and monitored longitudinally throughout the study period.^50^ The study was conducted between April and November 2025.

#### Assessments and endpoints

Assessments were performed at baseline and after 30, 60, and 90 days of treatment. The prespecified primary efficacy endpoint was the between-group difference in the change from baseline to day 90 in the A/G ratio, assessed by dual-energy X-ray absorptiometry (DXA). Additional body composition measures, including total fat mass, lean body mass, regional fat mass, body weight, body mass index, skinfold thickness, and anthropometric circumferences, were analyzed as secondary outcomes. Other secondary endpoints included glycemic parameters, insulin sensitivity indices, biochemical markers related to obesity, type 2 diabetes and cardiovascular risk, and inflammatory markers (IL-6, TNF-α, and high-sensitivity C-reactive protein). Safety was evaluated through adverse event monitoring, vital signs, and clinical laboratory assessments.

#### Body composition and fat distribution

Regional body composition was assessed by DXA at baseline and day 90 using a Lunar DXA system (GE Healthcare, Chicago, IL, USA).^51^^,^^52^ Images were analyzed using the manufacturer’s enCORE software (version 14.10), which automatically quantified total and regional lean and fat mass and calculated the A/G ratio.^53^ Because previous studies suggested that Pep19 preferentially affects regional adipose tissue distribution rather than total adiposity, the between-group difference in the change from baseline to day 90 in the A/G ratio was selected as the prespecified primary efficacy endpoint.^20^^,^^21^^,^^23^

Skinfold thickness was measured at seven anatomical sites (triceps, subscapular, midaxillary, suprailiac, abdominal, thigh, and pectoral) according to the Jackson and Pollock protocol^54^ using a validated scientific skinfold caliper.^45^^,^^55^ Waist, abdominal, hip, and chest circumferences were obtained using standardized anatomical landmarks, and body weight was measured with a calibrated digital scale under standardized conditions. All anthropometric assessments were performed by trained personnel blinded to treatment allocation.

#### Blood pressure and heart rate

Systolic and diastolic blood pressure and heart rate were measured after a 10-min seated rest using a validated automated device (Omron HEM-6232T, OMRON Healthcare Europe B.V., Hoofddorp, Netherlands).^56^

#### Clinical laboratory assessments

Fasting blood samples were collected in the morning after at least 8 hours of fasting. Routine biochemical parameters, including glucose, lipids, liver enzymes, and renal markers, alongside HbA1c and hematological indices, were analyzed using automated platforms validated according to the standards of the International Federation of Clinical Chemistry and Laboratory Medicine and the National Glycohemoglobin Standardization Program (using an automated platform Roche Cobas 8000®; São Paulo, SP, Brazil). Glycated hemoglobin (HbA1c) was quantified by low-pressure liquid chromatography (LPLC) using a Bio-Rad® system (Hercules, CA, USA). Hormones and inflammatory markers, specifically insulin, interleukin-6, tumor necrosis factor-alpha, thyroid-stimulating hormone, and free thyroxine, were quantified by electrochemiluminescence immunoassays (using an automated platform Roche Cobas 8000®; São Paulo, SP, Brazil; Roche, São Paulo, SP, Brazil). Insulin resistance was estimated using the homeostasis model assessment (HOMA) of insulin resistance (HOMA-IR); HOMA-IR = [fasting glucose (mg/dL) × fasting insulin (μIU/mL)]/405.^46^

#### Sleep quality assessment

Sleep quality was assessed using a 5-point numerical rating scale (NRS) with a Likert-type structure, based on participants’ subjective perception over the previous 30 days.^57^ Scores ranged from 1 (very poor) to 5 (very good), providing a direct measure of individual satisfaction with sleep.

A single-item NRS was selected to minimize participant burden and facilitate repeated assessments within the clinical trial setting, while capturing global perceived sleep quality. Although multi-component instruments such as the Pittsburgh Sleep Quality Index provide a more comprehensive evaluation of sleep domains, single-item sleep quality measures have been previously used and shown to correlate with multidimensional sleep assessments in clinical and epidemiological studies, supporting their validity as pragmatic patient-reported outcomes when sleep is not the primary endpoint.

#### Statistical analysis

All analyses were performed in R (v4.5.1) using the lme4 (v1.1-38), lmerTest (v3.2-1), emmeans (v1.10), car, and performance packages.^58^^,^^59^ Statistical significance was set at α = 0.05 (two-tailed) and mark with an asterisk (∗ means *p* < 0.05, ∗∗ means *p* < 0.01 and ∗∗∗ means *p* < 0.001), and 95% confidence intervals are reported for all contrasts (R script is available in supplemental information).

##### Baseline comparability (day 0)

Group balance at randomization was assessed by automatic test dispatch according to variable type. Continuous variables with within-group normality (Shapiro-Wilk *p* ≥ 0.05 in all three groups) were tested with one-way ANOVA followed by Tukey HSD pairwise comparisons. Continuous variables violating normality in at least one group were tested with the Kruskal-Wallis test followed by Dunn pairwise comparisons with Holm correction. Binary categorical variables (sex, physical-activity status) were tested with Fisher’s exact test. Sleep quality, recorded on a 0–5 ordinal scale with half-point increments, was treated as continuous. Descriptive statistics are reported as mean ± SD (normal), median [IQR] (non-normal), or *n* (%) (binary).

##### Primary analyses by outcome structure

Outcomes were grouped according to the number of post-baseline visits with available data, and analyses were dispatched accordingly.

*Longitudinal linear mixed-effects models (≥2 post-baseline visits)*: For each outcome measured at two or more post-baseline timepoints (day 30, 60, and/or 90), a linear mixed-effects model (LMM) was fitted with treatment group (placebo, 5 mg, 10 mg), visit (categorical), and their interaction as fixed effects. The participant-level day 0 value of the outcome, sex, and age (centered at the sample mean) were included as fixed-effect covariates. A by-participant random intercept accounted for within-subject correlation across visits. For outcomes expressed as percentage of baseline, the baseline covariate was omitted because the day 0 value is constant (100%) by construction. Models were fitted by restricted maximum likelihood (REML) with the bobyqa optimizer. Denominator degrees of freedom and *p*-values for fixed effects were obtained via the Satterthwaite approximation.

*ANCOVA (single post-baseline visit)*: For outcomes measured at a single post-baseline timepoint (day 90), including the DXA-derived A/G ratio, the longitudinal linear mixed models used for repeatedly measured outcomes were not applicable. The A/G ratio, which is expressed throughout the manuscript as a percentage of each participant’s own baseline, was analyzed on that percentage-of-baseline scale as the primary analysis, using a one-way model of the day-90 percentage adjusted for sex and centered age; because the baseline of a percentage-of-baseline variable is 100% by construction, no baseline covariate was entered. As supporting analyses, and to provide the absolute values requested during review, the day-90 value was also analyzed by analysis of covariance (ANCOVA) of the form post-treatment value ∼ group + sex + centered age + baseline value, on both the absolute scale and the log (multiplicative) scale, without random effects.

*Logistic ANCOVA-equivalent (binary two-timepoint outcome):* Self-reported physical-activity status (0 = inactive, 1 = active) could not be modeled with a longitudinal binomial GLMM because one or more groups exhibited no within-subject variability across visits, producing numerical separation. We therefore fitted a cross-sectional logistic regression of day 90 status adjusted for day 0 status: glm(activityday 90 ∼ group + sex + centered_age + activityday 0, family = binomial[link = “logit”]). This is the binary analogue of the ANCOVA described above. Wald-based type-III tests (car::Anova), odds ratios with 95% Wald confidence intervals, and estimated marginal means on the probability scale (emmeans, type = “response”) with Dunnett-adjusted contrasts versus placebo (many-to-one). The model was estimated by maximum likelihood.

##### Estimated marginal means and pairwise contrasts

Estimated marginal means (EMMs) were computed with the emmeans package. Because the trial compares two active doses against a single common placebo control, the family of comparisons of interest is many-to-one (each dose versus placebo); contrasts of each active dose versus placebo (within each visit for LMMs, and overall, for ANCOVA and logistic models) were therefore adjusted for multiple comparisons using the Dunnett method (contrast[emm, method = “trt.vs.ctrl”, ref = “Placebo”, adjust = “dunnett”]). 95% confidence intervals are reported for all contrasts.

##### Sensitivity analyses

Distributional assumptions were evaluated prior to model fitting. Within-cell normality was assessed by the Shapiro-Wilk test for every group × time combination, and homogeneity of variances at each visit by Levene’s test (median-centered). For outcomes with severe departures from normality-operationally defined as more than 50% of group × time cells with Shapiro-Wilk *p* < 0.05; sensitivity analyses re-fitted the same model after a log(1 + x) transformation applied to both the outcome and its baseline covariate. Results were considered robust when the significance of the group × time interaction (or group main effect, for two-timepoint analyses) and the pattern of pairwise contrasts were concordant between untransformed and log-transformed models. Both versions are reported side by side in the supplemental tables. In addition, for the primary endpoint (the between-group difference in the change from baseline to day 90 in the A/G ratio) we performed a sensitivity analysis of additive versus multiplicative scale structure, computed independently of the treatment contrast: the Box-Cox optimal power transformation (λ) was estimated with its 95% confidence interval; the mean-variance relationship was characterized through the coefficient of variation across group × time cells; and homoscedasticity was assessed with the Breusch-Pagan score test (carncvTest), comparing the additive (absolute-scale) model against the multiplicative (log-scale) model, complemented by Shapiro-Wilk tests of residual normality.

##### Model quality and diagnostics

For each LMM, intraclass correlation coefficients (adjusted and conditional) and marginal/conditional *R*^2^ were computed with the performance package.^58^^,^^59^ Residual diagnostics, QQ-plots, residuals versus fitted values, scale-location plots, and Shapiro-Wilk on residuals, were inspected for all primary endpoint models.

##### Exploratory cluster analysis of individual response profiles

To characterize inter-individual heterogeneity in treatment response, we performed an unsupervised *k*-means clustering analysis on day 90 outcome values expressed as a percentage of each participant’s own baseline (day 0 = 100%). Eight clinically relevant features spanning three metabolic domains were selected *a priori*: adiposity (fat mass, abdominal skinfold thickness, sum of skinfolds), glycemic control and insulin sensitivity (HbA1c, HOMA-IR), and inflammatory profile (IL-6, TNF-α). Features with more than 30% missing data at day 90 were excluded, and the analysis was restricted to participants with complete observations across all retained features. Features were standardized to *z*-scores prior to clustering to ensure equal contribution to the Euclidean distance metric.

The optimal number of clusters (*k*) was determined by jointly evaluating three complementary criteria: the within-cluster sum of squares (elbow method), the average silhouette width, and the gap statistic (*B* = 250 bootstrap replicates). *K*-means was then fitted with the selected *k* using 50 random initializations (nstart = 50) and a maximum of 250 iterations to ensure convergence. Cluster validity was assessed via per-observation silhouette widths; clusters with a mean silhouette width below 0.25 were flagged as poorly separated.

To evaluate whether the identified response phenotypes reflected pre-existing patient characteristics rather than treatment effects, baseline values of metabolic, anthropometric, and inflammatory variables were compared across clusters using the Kruskal-Wallis test for continuous variables and the chi-squared test for the distribution of treatment groups and sex. Clustering was performed with the cluster and facto extra packages in R (v4.5.1).

### Additional resources

This study was registered at the Brazilian Registry of Clinical Trials (UTN: U1111-1334-7473; RBR-10zchcdk): https://ensaiosclinicos.gov.br/rg/RBR-10zchcdk.

## Supplemental information

Document S1. Figure S1 and Table S1

Table S2. LMM_Results_Pep19_v5_dunnett.xlsx (comprehensive statistical analysis of all experimental datasets)

Table S3. dataset_pep19_deidentified.csv (anonymized raw data)

Data S1. LMM_analysis-vf-V5-dunnett.R (R script is used to analyzed all raw data)

## Footnotes

Supplemental information can be found online at https://doi.org/10.1016/j.isci.2026.117471.
