# Supplementary material for: Effects of Pep19 on adipose tissue distribution and metabolic parameters in adults with obesity: A randomized, double-blind, placebo-controlled trial
Source: iScience. 2026 Sep 8;29(10):117471. doi: 10.1016/j.isci.2026.117471 (PMC13572999; doi:10.1016/j.isci.2026.117471)
Supplement: Supplementary file 1 [file mmc1.pdf]

iScience, Volume *29*

Supplemental information

Effects of Pep19 on adipose tissue distribution and metabolic parameters in adults with obesity: A randomized, double-blind, placebo-controlled trial

Davi Vantini, João Roberto de Sá, Roseli O.S. Sarni, Sonia Hix, Fabio L. Filho, Raphael Ruiz, Ricardo A. Remer, Arnon Krongrad, Luiz Felipe Martucci, Emer S. Ferro, Andrea S. Heimann, and Fernando L.A. Fonseca

Supplementary Figure 1

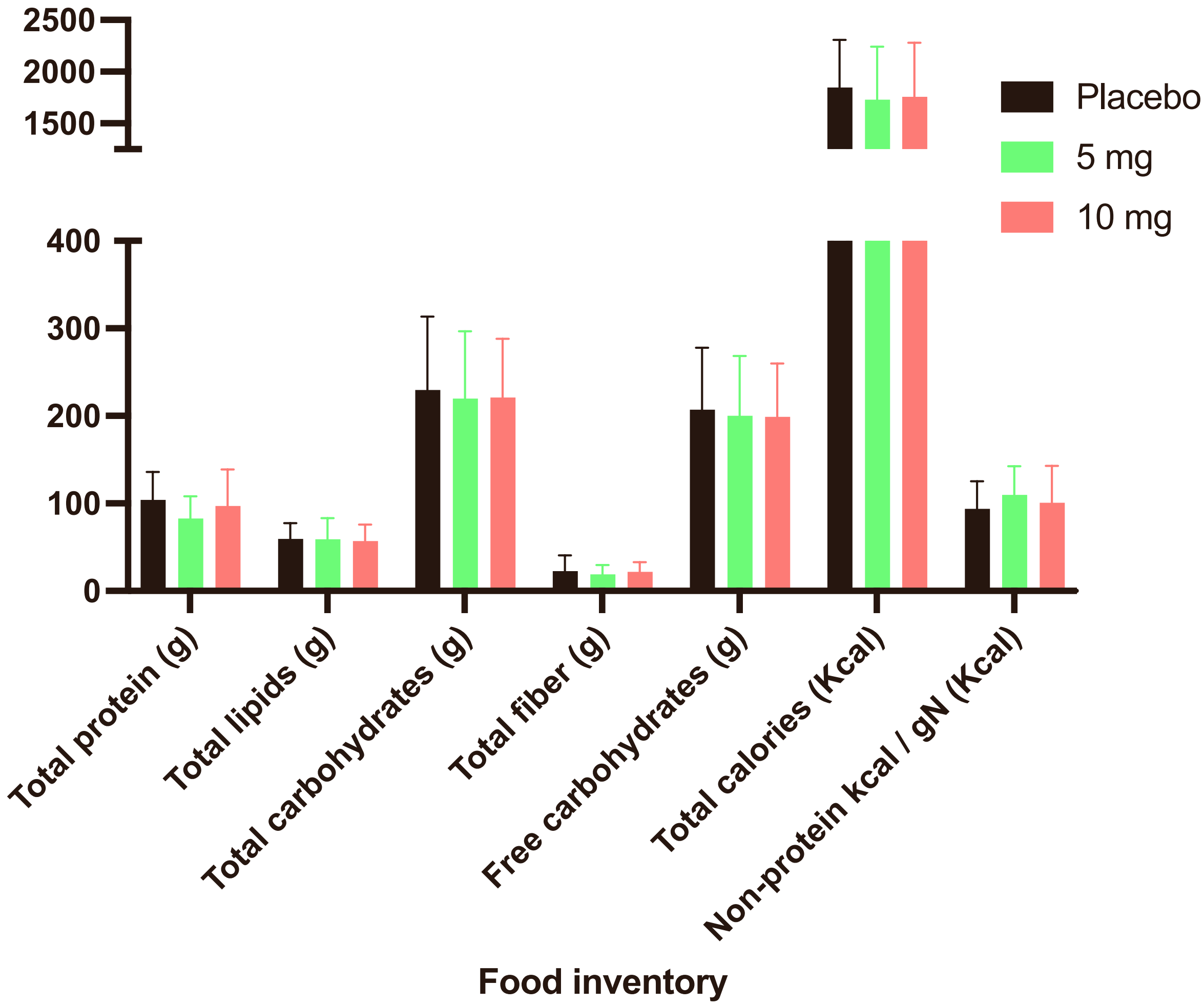

Supplemental Figure 1. Dietary intake across experimental groups. Total protein, total lipids, total carbohydrates, total fiber, free carbohydrates, total caloric intake and non-protein energy expressed as kcal per gram of nitrogen (kcal/gN) were evaluated in subjects treated with placebo, 5 mg Pep19 or 10 mg Pep19. Data are presented as mean ± standard deviation. Sample size: n = 18 (placebo); n = 20 (5 mg Pep19); n=18 (10 mg Pep19). Error bars indicate standard deviation of the mean. 5 mg and 10 mg refer to the respective Pep19 treatment cohorts. No significant differences were observed among groups for any of the analyzed dietary intake.

Supplementary Table – S1. Effects of Pep19 treatment on safety outcomes (tertiary endpoints) after intervention

Outcome	Placebo	5 mg	10 mg
**Endpoints in absolute values at 90 days**			
Safety outcomes			
Basophil (%)	73.61 ± 33.07	71.00 ± 36.84	59.11 ± 27.36
Iron concentration	1.20 [1.20–1.40]	1.20 [1.20–1.40]	1.30 [1.20–1.40]
Creatinine (mg/dL) — renal	0.89 ± 0.13	0.88 ± 0.21	0.84 ± 0.21
Erythrocytes (million/mm^3)	4.99 ± 0.47	5.12 ± 0.46	4.96 ± 0.56
Eosinophils (%)	22.00 [17.00–28.00]	22.50 [18.75–47.00]	17.50 [15.25–28.00]
Free thyroxine T4 (ng/dL)	1.26 ± 0.53	1.22 ± 0.34	1.18 ± 0.18
Hematocrit (%)	42.54 ± 3.74	43.20 ± 4.12	41.94 ± 5.12
Hemoglobin (g/dL)	14.45 ± 1.20	14.52 ± 1.48	14.06 ± 1.72
Thyroid-stimulating hormone (µUI/mL)	1.88 [1.48–2.21]	1.92 [1.62–2.69]	1.84 [1.36–2.29]
Lymphocytes (%)	3314.83 ± 810.20	3061.00 ± 652.61	3111.22 ± 813.72
Mean corpuscular hemoglobin (pg)	29.07 ± 1.47	28.30 ± 1.38	28.50 ± 1.54
Mean corpuscular hemoglobin conc. (g/dL)	33.91 ± 1.21	33.40 ± 1.27	33.72 ± 1.02
Monocytes (%)	681.17 ± 200.86	757.00 ± 187.90	696.06 ± 221.92
Mean platelet volume MPV (μm^3)	10.20 ± 1.26	9.64 ± 0.74	9.99 ± 0.74
Platelets (mm^3)	280.54 ± 48.17	292.25 ± 52.36	270.93 ± 49.73
Red Cell Distribution Width RDW (%)	12.68 ± 0.77	13.02 ± 0.98	13.20 ± 0.90
Segmented neutrophils (%)	56852.78 ± 8564.13	58295.00 ± 9337.39	53985.56 ± 13319.58
Thyroid-stimulating hormone TSH (µUI/mL)	1.97 [1.64–2.24]	1.88 [1.50–2.69]	2.04 [1.45–2.36]
Total leukocytes (%)	7133.33 ± 1782.26	7320.00 ± 1819.66	8061.11 ± 1973.99
Urea (mg/dL) — renal	30.36 ± 7.63	31.77 ± 8.72	27.48 ± 7.70
Uric Acid (mg/dL) — renal	5.36 ± 1.16	5.46 ± 1.90	5.54 ± 1.46
**Endpoints in % of baseline at 90 days**			
Basophil — % change from baseline	6.15 [81.15–115.00]	100.00 [79.64–110.39]	117.14 [100.00–138.33]
Iron concentration — % change from baseline	96.82 ± 12.83	100.31 ± 14.38	98.92 ± 11.86
Creatinine — % change from baseline	104.55 ± 12.56	101.66 ± 17.88	97.52 ± 8.82
Erythrocytes — % change from baseline	99.82 ± 10.39	103.31 ± 5.47	99.98 ± 4.24
Eosinophils — % change from baseline	109.09 [95.45–128.57]	121.39 [98.08–167.19]	109.09 [100.00–123.08]
Free T4 — % change from baseline	107.06 ± 48.21	92.08 ± 12.30	93.71 ± 19.22
Hematocrit — % change from baseline	100.78 ± 3.94	101.83 ± 5.74	99.37 ± 4.95

Outcome	Placebo	5 mg	10 mg
Hemoglobin — % change from baseline	99.31 ± 3.09	100.39 ± 5.19	97.78 ± 4.11
TSH — % change from baseline	107.67 ± 43.70	123.03 ± 72.51	93.64 ± 28.85
Lymphocytes — % change from baseline	103.03 ± 19.87	93.62 ± 11.50	103.78 ± 41.74
MCH — % change from baseline	97.74 [96.58–100.00]	98.33 [96.32–100.00]	98.61 [96.67–100.00]
MCHC — % change from baseline	98.37 ± 4.01	98.01 ± 3.38	98.85 ± 3.23
Monocytes — % change from baseline	93.59 ± 16.75	102.39 ± 14.47	103.93 ± 30.91
MPV — % change from baseline	97.75 ± 6.83	98.14 ± 6.60	96.87 ± 5.24
Platelets — % change from baseline	105.14 ± 14.68	100.07 ± 11.59	96.27 ± 10.97
RDW — % change from baseline	98.91 ± 2.61	99.72 ± 3.98	101.08 ± 3.59
Segmented neutrophils — % change from baseline	97.42 [92.71–103.34]	105.44 [98.10–110.73]	99.10 [95.63–103.45]
TSH (alt) — % change from baseline	111.31 ± 42.14	123.38 ± 77.76	95.91 ± 30.65
Total leukocytes — % change from baseline	99.70 ± 22.46	97.94 ± 19.38	95.93 ± 17.72
Urea — % change from baseline	106.53 ± 28.85	104.50 ± 26.41	107.74 ± 23.46
Uric Acid — % change from baseline	95.37 ± 8.72	93.33 ± 23.30	90.71 ± 9.51

Supplementary Tables – S1. Effects of Pep19 treatment on tertiary endpoints after intervention. Absolute end-of-study values and percentage of baseline are shown for participants receiving placebo, Pep19 5 mg, or Pep19 10 mg. Continuous variables are presented as mean ± standard deviation (SD) for normally distributed data or median [interquartile range] for non-normally distributed data. Percentage from baseline was calculated for each participant and summarized by treatment group. No statistically significant within-group differences relative to baseline were observed across any of the evaluated parameters.
